# Mediastinal Extralobar Pulmonary Sequestration Concurrent With Spontaneous Pneumothorax: A Case Report of Difficult Preoperative Diagnosis

**DOI:** 10.7759/cureus.72959

**Published:** 2024-11-04

**Authors:** Kensuke Takei, Yusuke Takanashi, Motohisa Shibata, Keigo Sekihara, Kazuhito Funai

**Affiliations:** 1 First Department of Surgery, Hamamatsu University School of Medicine, Hamamatsu, JPN

**Keywords:** extralobar sequestration, mediastinal tumor, pericardial defect, pneumothorax, thoracic surgery

## Abstract

Extralobar pulmonary sequestration (EPS) in the mediastinum is rare, and preoperative diagnosis can be challenging. We report a case of EPS in the middle mediastinum, where a congenital pericardial defect became apparent on computed tomography (CT) imaging as pneumopericardium concurrent with spontaneous pneumothorax. The patient presented with a left spontaneous pneumothorax. A chest plain CT revealed bullae in the apical segment of the left upper lobe and the S6 segment of the lower lobe, a 3.2 cm solid mass in the left middle mediastinum, and a pericardial defect with pneumopericardium beside the main left pulmonary artery. We initially considered the mediastinal mass to be a hematoma resulting from the rupture of adhesions between the lung and mediastinum due to the onset of spontaneous pneumothorax. We performed a bullectomy using video-assisted thoracoscopic surgery. Intraoperative findings revealed that the mediastinal mass was not a hematoma but solid tissue, raising suspicion of a mediastinal tumor. We transected the feeding artery and drainage vein using a vessel sealing system and resected the mediastinal mass. Histopathological examination revealed bronchial structures and lung parenchyma, confirming a diagnosis of EPS. The pericardial defect manifesting as pneumopericardium due to concomitant pneumothorax could have provided clues to an accurate preoperative diagnosis of EPS.

## Introduction

Extralobar pulmonary sequestration (EPS) is a rare congenital lung disease that accounts for approximately 15% of pulmonary sequestrations [1]. Most cases of EPS occur between the left lower lobe and the diaphragm [2]. EPS generally becomes apparent in early infancy and is often accompanied by various malformations [3]. However, rare cases of EPS located in the mediastinum with subtle comorbid malformation have also been reported; in such cases, preoperative diagnosis can be challenging, and the surgeon may be compelled to perform interventions during the surgery [4,5]. Here, we report a case of EPS in the middle mediastinum, where a congenital pericardial defect, a comorbid malformation, became apparent on computed tomography (CT) imaging as pneumopericardium concurrent with spontaneous pneumothorax. This case study highlights the lessons learned from instances where resection was performed without diagnosing EPS based on preoperative and intraoperative findings, and EPS was subsequently identified through postoperative pathological diagnosis.

## Case presentation

**Figure 1:**
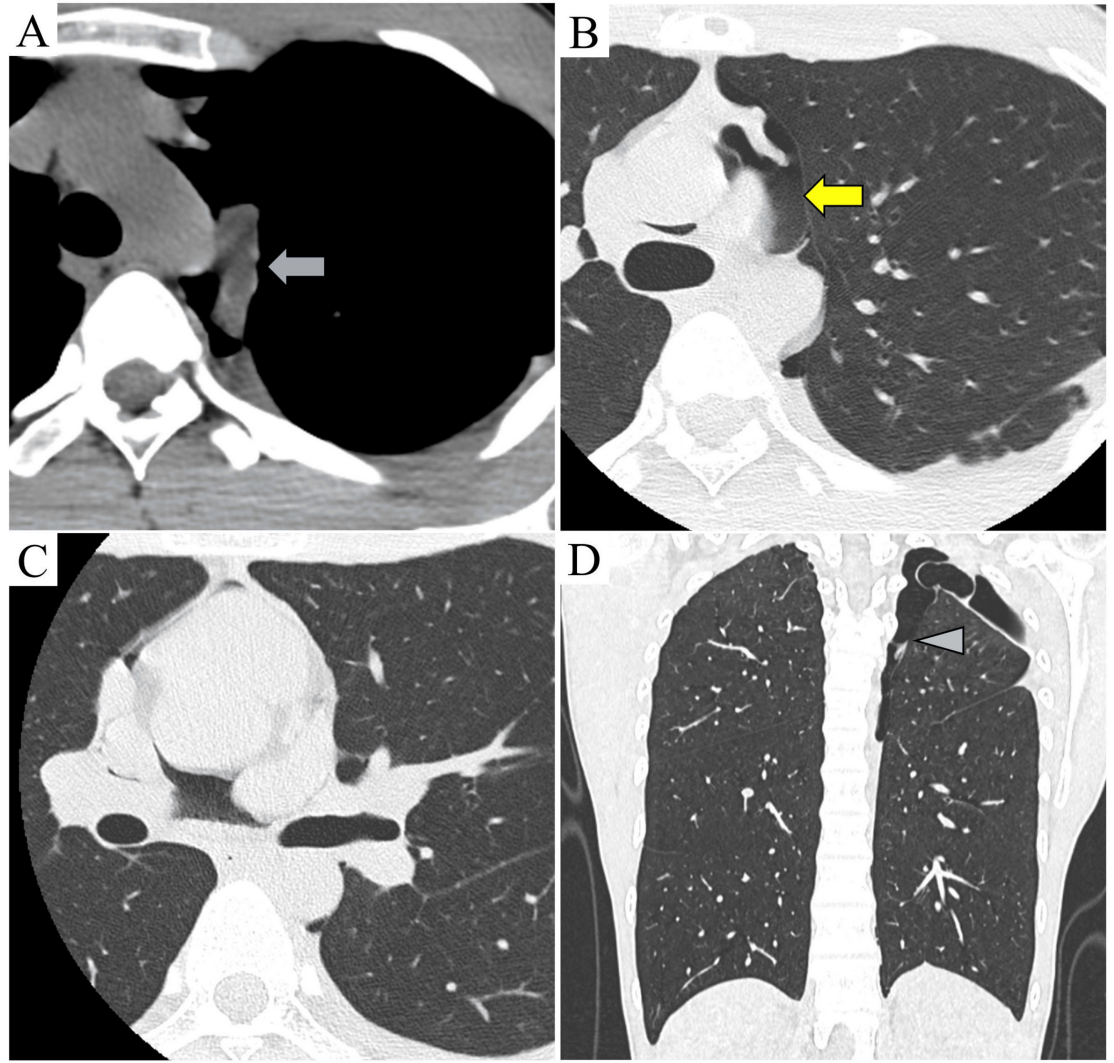
Radiological images. (A) Preoperative plain computed tomography (CT) revealed a 3.2 cm solid mass in the left middle mediastinum (blue arrow). (B, C) Axial CT revealed a pericardial defect (yellow arrow) with pneumopericardium. (D) Coronal CT revealed a left pneumothorax, a bulla in the apical segment of the left upper lobe, and adhesions (arrow head).

A 19-year-old man, who had never smoked, presented with chest pain. He was diagnosed with a left spontaneous pneumothorax, which improved with observation. Three months after the first visit, a recurrence of the left pneumothorax was observed on chest radiography at the annual medical examination. A chest plain CT revealed bullae in the apical segment of the left upper lobe and the S6 segment of the lower lobe. In addition, a 3.2 cm solid mass in the left middle mediastinum, and a pericardial defect with pneumopericardium beside the main left pulmonary artery were observed (Figures 1A-D). We preoperatively considered the mass to be a hematoma resulting from the rupture of adhesions between the lung and mediastinum due to the onset of spontaneous pneumothorax. Thus, we planned bullectomy under video-assisted thoracoscopic surgery (VATS) for the spontaneous pneumothorax.

**Figure 2:**
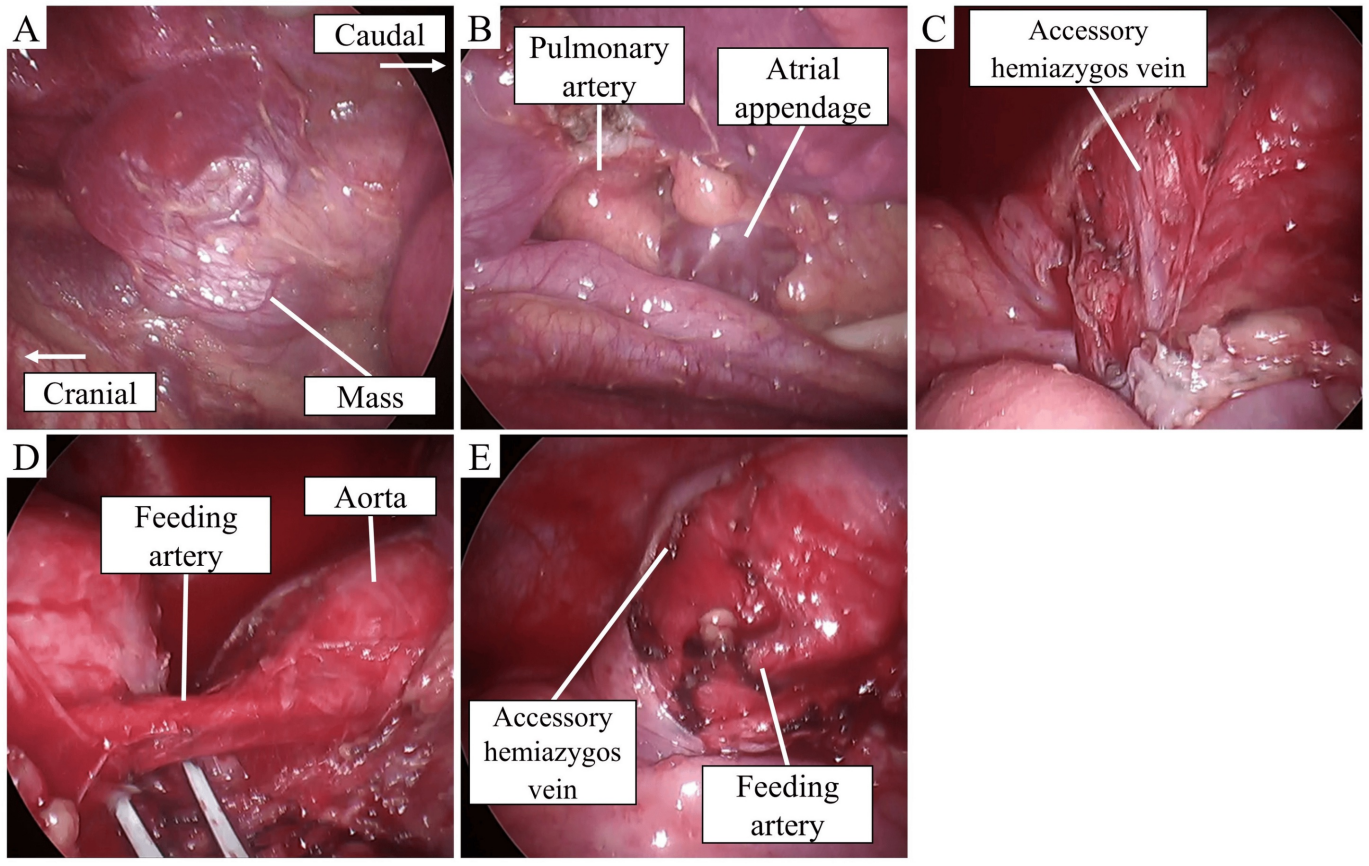
Intraoperative findings. (A) The middle mediastinal mass presented as solid tissue contiguous with the pleura covering the aortic arch, containing white cystic and dark red fan-shaped regions. (B) The pulmonary artery and left atrial appendage were exposed due to pericardial defect. (C) Drainage vein into the accessory hemiazygos vein from the mediastinal mass. (D) The feeding artery to the mediastinal mass. (E) The feeding artery and drainage vein were closed using a vessel sealing system.

We initiated VATS with three ports in the right lateral position. We resected the bullae in the apex of the upper lobe and the S6 segment of the lower lobe, respectively, using ECHELON™+ Stapler (Ethicon, Inc., Cincinnati, Ohio). Next, we observed the mediastinal mass, preoperatively considered to be a hematoma based on CT findings. Contrary to our expectations, the mediastinal mass was not a hematoma but a solid tissue contiguous with the pleura covering the aortic arch, containing white cystic and dark red fan-shaped regions (Figure 2A). The pericardium dorsal to the hilum was also elliptically deficient, exposing the main pulmonary artery and the left atrial appendage (Figure 2B). The differential diagnosis for the mediastinal mass included a solitary fibrous tumor originating from the mediastinal pleura or a bronchogenic cyst; thus, we decided to resect the mediastinal mass. In the resection of the mediastinal mass, we transected the aberrant feeding artery, 3 mm in diameter, and drainage vein into the accessory hemiazygos vein using the LigaSure™ vessel sealing system (Medtronic, Dublin, Ireland) (Figures 2C-E). At the time of arterial transection, the origin of the feeding artery was not recognized. After complete resection, we confirmed the absence of malignancy based on intraoperative rapid diagnosis. The patient was discharged without postoperative complications.

**Figure 3:**
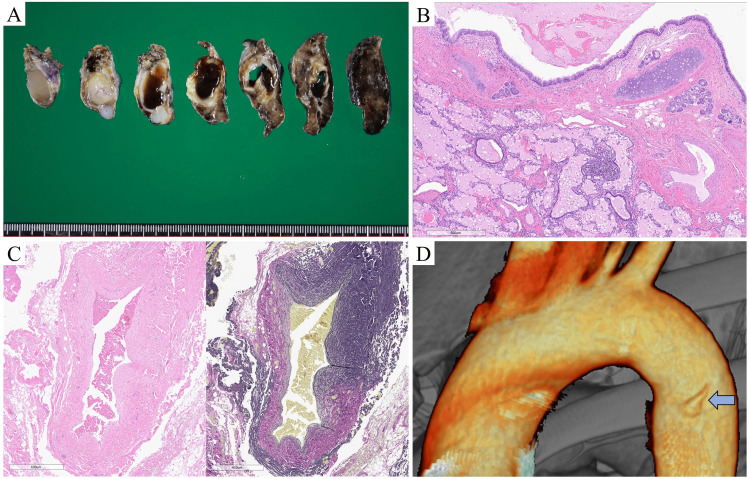
Pathological findings and three-dimensional computed tomography image. (A) Macroscopic findings. The mass consisted of a mucin-filled bronchial cyst. (B) Hematoxylin and eosin (HE) stained image. Histopathological examination revealed dilated bronchial structures with cartilage and glands, and lung parenchymal tissue associated with edema (scale bar, 600 µm). (C) HE and Elastica van Gieson (EVG) stained images. Histopathological examination revealed an elastic feeding artery (scale bar, 600 µm). (D) Postoperative three-dimensional computed tomography revealed an aberrant feeding artery stump from the descending aorta (arrow).

Macroscopic examination of the surgical specimen revealed that the mass contained a mucin-filled bronchial cyst (Figure 3A). Histopathological examination revealed dilated bronchial structures with cartilage and glands, lung parenchymal tissue associated with edema, and elastic feeding artery (Figures 3B, C). No malignancies were observed. Based on these histopathological findings, the patient was diagnosed with EPS. Postoperative contrast-enhanced CT was performed to evaluate the feeding artery stump. A feeding artery stump, 5 mm in length, from the descending aorta was detected on a three-dimensional CT (Figure 3D). The patient is under follow-up without any symptoms.

## Discussion

EPS is separated from the normal lung by its pleural covering and has no connection to the tracheobronchial tree [3]. EPS is also associated with other congenital malformations [3]. The standard treatment for EPS is surgical resection [6], which requires careful transection of the aberrant artery. The lessons learned from this case were that the preoperative diagnosis of mediastinal EPS was difficult, and as a result, we were not prepared to recognize and safely manage the aberrant feeding artery originating from the aorta during the surgery. We discuss the reasons for preoperative diagnostic difficulties and aberrant arterial transection as follows.

We discuss the reasons for the preoperative diagnostic difficulty of EPS from three perspectives: lesion localization, diagnostic imaging, and concurrent malformations. Regarding the localization of the EPS, most cases occur between the left lower lobe and diaphragm [2], and middle mediastinal localization, like our case, is rare [4]. Because distinguishing EPS in the mediastinum from mediastinal tumors is challenging, diagnosis of EPS can be made intraoperatively or postoperatively [4,5]. As for diagnostic imaging, chest CT in cases of EPS shows the lesion as a homogenous and well-circumscribed soft tissue density mass [3]. Aberrant arteries and the drainage veins into the systemic veins can often be identified by utilizing contrast enhancement [3]. In this case, we suspected recurrent pneumothorax preoperatively and performed only plain CT. As a result, we were unable to preoperatively identify an aberrant feeding artery originating from the aorta. EPS is associated with congenital malformations such as congenital cystic adenomatoid malformation, congenital diaphragmatic hernia, lung and chest wall deformities, vertebral deformities, foregut malformations, bronchogenic cysts, and complex cardiac anomalies [6]. Pericardial defects with concurrent EPS are rare and limited to several reports [7-10]. In this case, the physical examination revealed chest pain due to spontaneous pneumothorax, with no other symptoms related to EPS or a pericardial defect. The pericardial defect manifested as pneumopericardium due to pneumothorax. However, due to its rarity, we could not recognize the pericardial defect as a comorbid malformation with the EPS. To summarize, the reasons for the preoperative misdiagnosis are considered as follows: EPS was not considered in the differential diagnosis of a middle mediastinal mass, and only plain CT was performed as a preoperative examination for spontaneous pneumothorax, leading to the failure to identify the aberrant artery and drainage vein and the pericardial defect was not recognized as a rare comorbid malformation of EPS.

In this case, we transected the feeding artery using a vessel sealing system, because we did not recognize the vessel as an aberrant artery of the EPS originating from the aorta. After EPS was diagnosed through histopathological examination, a review of the surgical video and a contrast-enhanced CT scan were conducted, which revealed that the feeding artery originated from the aorta. Aberrant arteries in pulmonary sequestration and anomalous systemic arterial supply to the basal lung are transected using various surgical techniques, including ligation, suturing, and stapler use [11,12]. In our case, the aberrant artery with a diameter of 3 mm would not be suitable for stapling due to its small size. The vessel sealing system is appropriate because it can effectively seal vessels and vascular bundles up to 7 mm in diameter [13]. However, the walls of aberrant arteries show pathological changes such as fibrous intimal thickening, atherosclerosis, and chronic inflammation, which may underlie future aortic problems [12]. In addition, a concern regarding aneurysm formation on the stump of an aberrant artery is reported [14]. Thus, it may be argued that the vessel sealing system alone is insufficient to transect the aberrant artery. In this case, ligating at the aberrant artery's root or suturing the stump would have been preferable. Careful follow-up with contrast-enhanced CT is needed to check for stump aneurysm formation.

## Conclusions

The preoperative diagnosis of middle mediastinal EPS is difficult. Preoperative suspicion of EPS can result in safe handling of aberrant arteries. In our case, the pericardial defect, which manifested as pneumopericardium due to concomitant spontaneous pneumothorax, could have provided clues to an accurate preoperative diagnosis of EPS.
